# Is It Safe to Reduce Inhaled Corticosteroid Dose in Severe Asthma Patients Receiving Biologic Therapy?

**DOI:** 10.1002/clt2.70204

**Published:** 2026-10-22

**Authors:** Nagihan Orhan Özer, Betül Özdel Öztürk, Begüm Görgülü Akın, Ceren Kaplankıran, Şadan Soyyiğit

**Affiliations:** ^1^ Department of Immunology and Allergy Ankara Bilkent City Hospital Ankara Turkey; ^2^ Department of Immunology and Allergy Medical Faculty of Ankara Yildirim Beyazit University Ankara Turkey

**Keywords:** asthma control, biologic therapy, inhaled corticosteroids, severe asthma, step‐down treatment

## Abstract

**Background:**

Biologic therapies can control severe asthma and may modify its natural history beyond symptom control. However, step‐down is not standardized, and its safety has not been clearly established.

**Methods:**

This single‐center retrospective cohort study reviewed adults with severe asthma on biologics (January 2019–May 2024). Step‐down strategies (discontinuation of add‐on therapy and/or inhaled corticosteroid [ICS] dose reduction) were described, success rates calculated, and clinical characteristics compared between groups with and without step‐down.

**Results:**

Fifty‐one patients were included, of whom 10 (19.6%) received omalizumab, 34 (66.7%) mepolizumab, and 7 (13.7%) benralizumab. After excluding 6 patients unsuitable for step‐down assessment, 45 patients were compared. Step‐down was attempted in 21 of these (46.7%) and succeeded in 17 (81.0%), defined as no systemic corticosteroid use, emergency visit, loss of control (ACT < 20), or exacerbation through the last visit. The most frequent interventions were ICS dose reduction (*n* = 7, 33.3%) and montelukast discontinuation (*n* = 7, 33.3%). Biologics had been received for a mean of 42.6 ± 14.0 months, and the reduced regimen was maintained for 8.15 ± 5.23 months. Compared with the non–step‐down group, the step‐down group had a higher proportion of males (*p* = 0.005) and longer follow‐up (*p* = 0.007).

**Conclusion:**

In this real‐world cohort, step‐down was feasible and appeared safe, with most patients maintaining the reduced regimen without deterioration in control or exacerbations. These findings support supervised treatment minimization in carefully selected, well‐controlled patients, adding real‐world evidence to a limited literature.

## 1 Introduction

Asthma is a chronic disease characterized by variable airway inflammation with different pathophysiological mechanisms, symptoms, and progression [1]. Asthma that cannot be controlled despite high‐dose inhaled corticosteroid (ICS)‐long‐acting beta‐2 agonist (LABA) therapy or worsens when the dose is reduced is defined as severe asthma [2]. Patients with severe asthma are candidates for biologics. Biologics approved by the US Food and Drug Administration (FDA) and the European Medicines Agency (EMA) include omalizumab (anti‐IgE); mepolizumab, benralizumab, and reslizumab (anti–interleukin‐5/IL‐5R); dupilumab (anti–IL‐4R *alpha*); and tezepelumab (anti–thymic stromal lymphopoietin [TSLP]) [3, 4, 5, 6, 7, 8, 9, 10]. Real‐world studies and randomized trials have shown these agents improve symptoms, reduce exacerbations, emergency visits, and hospitalizations, and decrease oral corticosteroid (OCS) use [11, 12, 13, 14, 15]. In the treatment of severe asthma, step‐down approaches are important when feasible, particularly in patients receiving high‐dose corticosteroids or biologic therapies [1]. However, data on step‐down strategies with biologics remain limited. While evidence exists for OCS reduction, information on discontinuing additional therapies and lowering ICS doses is insufficient. Early evidence on step‐down treatment came largely from studies of omalizumab. A 2014 Cochrane review of patients with moderate to severe allergic asthma found that omalizumab allowed a significant reduction in ICS dose and reduced asthma exacerbations and hospitalizations compared with placebo [16]. A recent study showed that 92% of patients with severe eosinophilic asthma treated with benralizumab between 2019 and 2023 reduced their ICS dose, and 96% maintained it for 48 weeks without worsening symptoms or exacerbations [17]. This study aimed to evaluate the feasibility, safety, and outcomes of step‐down approaches, including discontinuing additional treatments and reducing ICS doses, in patients with severe asthma receiving biologics in our clinic.

## 2 Materials and Methods

### 2.1 Study Design

This study was designed as a retrospective cohort study which was approved by the Ankara Bilkent City Hospital No. 1 Scientific and Ethical Review Board for Medical Research (Decision No: TABED 1/377/2024, Date: 03/07/2024).

### 2.2 Data Recording and Study Group

The study data were obtained from the database of Ankara Bilkent City Hospital. The medical records of all consecutive adult patients diagnosed with severe asthma and receiving biologics at the Immunology and Allergy Diseases Clinic of Ankara Bilkent City Hospital between January 2019 and May 2024 were evaluated. Patients were not selected on the basis of follow‐up duration or treatment outcome, thereby minimizing the risk of selection bias. Given the retrospective nature of the study and the small patient population, no formal sample size calculation was performed. Instead, all consecutive patients meeting the inclusion criteria during the study period were included in the analysis. Confidence intervals for the main proportions were calculated using the Wilson method.

In all patients, asthma had been diagnosed by an allergy/immunology or pulmonology specialist according to Global Initiative for Asthma (GINA) criteria, based on a compatible clinical history together with objective demonstration of variable expiratory airflow limitation (bronchodilator reversibility, defined as an increase in the forced expiratory volume in 1 second (FEV1) of ≥ 12% and ≥ 200 mL) [1]. All patients had an established, longstanding diagnosis of asthma prior to enrollment.

Inclusion criteria were as follows:

- Age between 18 and 80 years,
- A confirmed diagnosis of severe asthma,
- Treatment with biologic agents (the biologic agents available at our clinic at the time of enrollment were omalizumab, mepolizumab, and benralizumab),
- No regular daily or alternate‐day maintenance OCS use at any time during the 12 months preceding the step‐down attempt.

Exclusion criteria included:

- Discontinuation of biologic therapy due to lack of clinical response,
- A confirmed diagnosis of Chronic Obstructive Pulmonary Disease (COPD)/asthma‐COPD overlap.

The collected data included sociodemographic and phenotypic characteristics, allergic and systemic comorbidities, smoking habits, age at asthma onset, disease duration, blood eosinophil levels, asthma phenotypes (allergic, eosinophilic, etc.), and biologic drugs used [1, 18].

Clinical follow‐up data included follow‐up duration in our clinic, GINA treatment steps and asthma medications at the beginning and end of follow‐up, Asthma Control Test (ACT) results, and discontinuation of add‐on treatments or attempts to reduce ICS dosage during follow‐up [1].

### 2.3 Definitions and Outcome Measurements

All patients included in the study underwent a comprehensive phenotypic assessment before initiation of biologic therapy to characterize the underlying asthma phenotype and guide biologic treatment selection [1]. Individuals with a history of allergy (atopic dermatitis, allergic rhinitis, food/drug allergy) and positive prick/intradermal tests or elevated serum specific immunoglobulin E (sIgE) levels were classified as having allergic asthma. In patients with elevated blood eosinophil counts, eosinophil levels were re‐measured at follow‐up visits (at intervals of 1 week to 1 month); patients with values > 150 cells/μL on at least three measurements were classified as having eosinophilic asthma.

Since Fractional Exhaled Nitric Oxide (FeNO) and sputum eosinophilia tests are not available in our clinic, they could not be used for phenotyping. sIgE was measured with the Immulite 2000 system (Siemens Healthcare Diagnostics); values ≥ 0.35 kU/L were considered positive. Skin prick testing was performed using commercial allergen extracts (ALK, Abello, Madrid, Spain; Lofarma, Milan, Italy) following European Academy of Allergy and Clinical Immunology (EAACI) guidelines. Antihistamines were discontinued 5–7 days before testing. A wheal ≥ 3 mm larger than the negative control was considered positive [19, 20].

At each visit, medication adherence, comorbidities, and environmental exposures were assessed. Symptom control was assessed using the ACT, with a score < 20 indicating inadequate control; exacerbation frequency, systemic corticosteroid requirement, and lung function were also taken into account, in line with the ERS/ATS criteria [21]. Those initiating biologic therapy were monitored monthly. ACT scores, inhaler technique, and comorbidities were reviewed at every visit. Spirometry was performed annually or when symptoms worsened. Because part of the study period coincided with the COVID‐19 pandemic, special precautions were implemented to ensure continuity of care. Patients receiving biologic therapy continued their scheduled monthly follow‐up visits, and evaluations were performed in isolation examination rooms when necessary. The pandemic did not influence patient selection, and no apparent deterioration in asthma control attributable to SARS‐CoV‐2 infection was observed in the study cohort.

Patients treated with biologics for ≥ 12 months were evaluated for an ACT score > 19 and the absence of asthma exacerbations, OCS use, and asthma‐related emergency visits or hospitalizations in the previous year. If all these criteria were met, additional medications were tapered or ICS doses were reduced. Spirometry was performed before and 6 months after step‐down intervention. Pulmonary function was measured with the COSMED micro Quark Spirometer (Italy), in accordance with American Thoracic Society and European Respiratory Society 2005 standards [22].

### 2.4 Evaluation of Treatment Reduction

Step‐down interventions were applied to patients who met the above eligibility criteria. Of the seven benralizumab‐treated patients, five were excluded from the between‐group comparison because they had received biologic therapy for less than 12 months. The remaining two, although treated for ≥ 12 months, did not undergo step‐down (one owing to a recent exacerbation and one to an ACT score < 20); consistent with the definition of the non–step‐down group, these two patients were included in that group. One patient initially classified as non‐step‐down, in whom dose reduction was performed only at the very end of the study period, was also excluded, as she represented neither an evaluable step‐down case nor a true non‐step‐down case. The step‐down comparison therefore included 45 patients (21 with and 24 without step‐down).

As the first step, in patients who were receiving add‐on controller therapy, long acting muscarinic antagonist (LAMA) and/or montelukast were discontinued (LAMA first, followed by montelukast), followed by stepwise ICS dose reduction (high → medium → low) [1]. Dose tapering was not done in patients already on low‐dose ICS or ICS/LABA (Figure 1).

**FIGURE 1:**
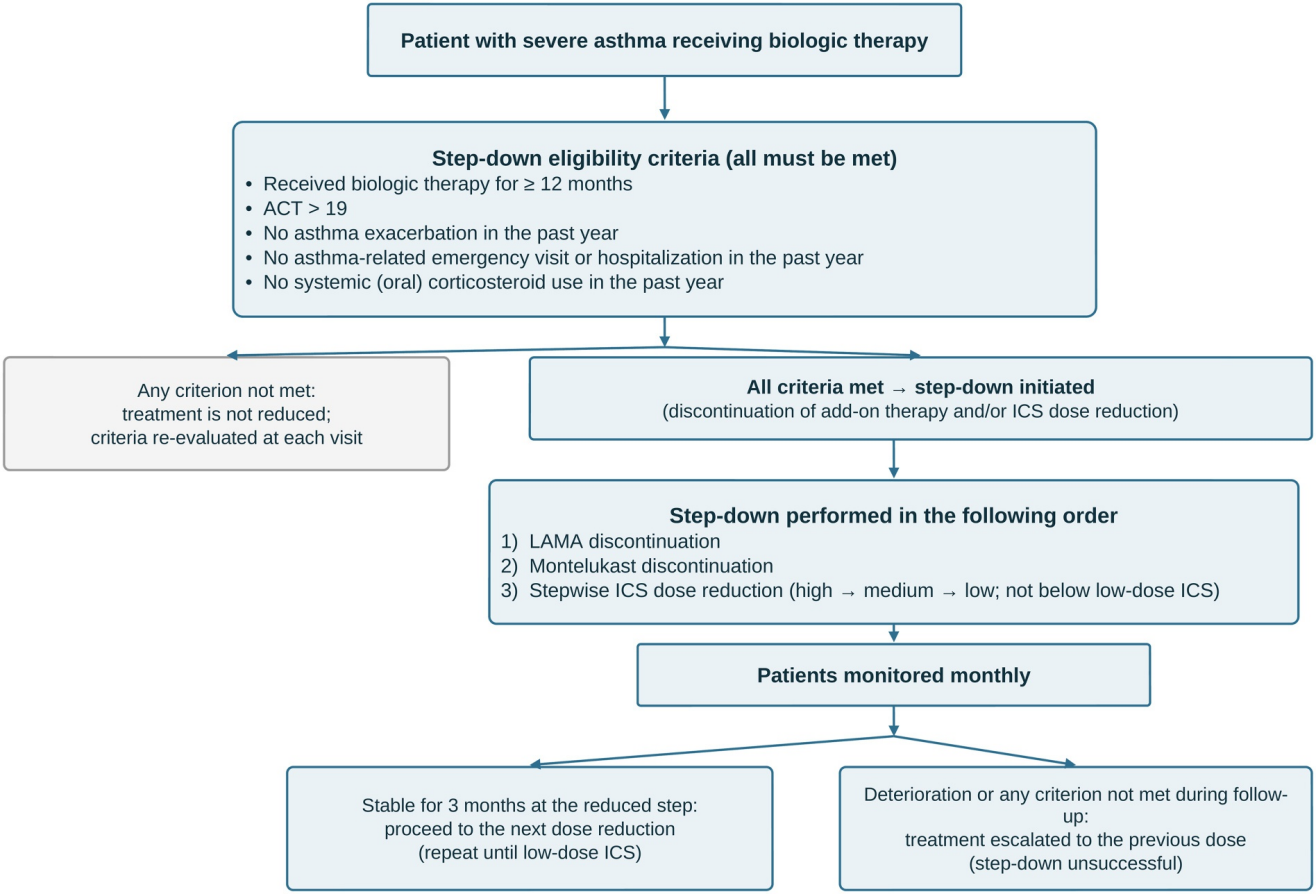
ICS dose reduction and discontinuation of additional treatments algorithm.

Patients were monitored monthly. Step‐down was considered successful when the reduced regimen was maintained throughout follow‐up without symptom deterioration, increased rescue medication (short‐acting beta‐2 agonist or as‐needed low‐dose ICS–formoterol used for symptom relief) use, lung function decline, or exacerbation. Conversely, step‐down was considered unsuccessful when any of these occurred and treatment had to be escalated to the previous dose. In this study, a clinically relevant decline in lung function was defined a priori as a fall in FEV1 of ≥ 10% from the pre–step‐down baseline. Exacerbations were defined according to GINA as a worsening of asthma requiring systemic corticosteroid use and/or an asthma‐related emergency department visit or hospitalization [1]. No minimum follow‐up duration was required for study inclusion, since all consecutive eligible patients were included.

### 2.5 Statistical Analysis

Statistical analyses were performed using IBM SPSS Statistics for Windows, version 26.0 (IBM Corp., Armonk, NY, United States). Descriptive statistics summarized demographic and clinical characteristics. Normality of continuous variables was assessed with the Shapiro–Wilk test. Normally distributed continuous variables were expressed as mean ± standard deviation (SD) and compared with the independent‐samples *t*‐test, whereas non‐normally distributed variables were expressed as median (minimum–maximum) and compared with the Mann–Whitney *U* test. Categorical variables were expressed as frequencies and percentages and compared with the Pearson chi‐square test; Fisher's exact test (or the Fisher–Freeman–Halton exact test) was used when expected cell counts were below 5. For within‐subject comparisons of the same patients at two time points (step‐down visit vs. last visit), the paired‐samples *t*‐test was used (or the Wilcoxon signed‐rank test for non‐normally distributed variables). For proportions, 95% confidence intervals were calculated using the Wilson score method. A two‐tailed *p* < 0.05 was considered statistically significant.

## 3 Results

### 3.1 Patient Characteristics

A total of 51 patients with severe asthma were included. Ten (19.6%) received omalizumab, 34 (66.7%) mepolizumab, and 7 (13.7%) benralizumab (Table 1). The mean age was 47.31 ± 12.77 years and 29 patients (56.9%) were female. The mean body mass index (BMI) was 27 ± 4.75. Asthma onset occurred at a mean age of 32.94 ± 12.39 years, with an average disease duration of 14.37 ± 7.48 years and follow‐up of 33 (6–72) months. Twenty‐nine patients (56.9%) were non‐smokers, 18 (35.3%) were ex‐smokers, and 4 (7.8%) were current smokers. Among patients with a history of smoking (*n* = 22), the cumulative smoking exposure was a median of 15 pack‐years (minimum–maximum: 8–28).

**TABLE 1:** Demographic and clinical characteristics of the patients (*n* = 51).

Characteristic	Category	Value
Gender, *n* (%)	Female	29 (56.9)
	Male	22 (43.1)
Age, years (mean ± SD)	47.31 ± 12.77	
Blood eosinophilia, *n* (%)	Eosinophilic (≥ 300 cells/μL)	43 (84.3)
Non‐eosinophilic (< 300 cells/μL)	8 (15.7)
Phenotype, *n* (%)	Allergic eosinophilic	20 (39.2)
	Non‐allergic eosinophilic	23 (45.1)
	Allergic non‐eosinophilic	8 (15.7)
Systemic comorbidity, *n* (%)	Hypertension	12 (23.5)
	Diabetes mellitus	3 (5.9)
	Ischemic heart disease	2 (3.9)
	Heart failure	1 (2.0)
	Hypothyroidism	5 (9.8)
	Other^a^	6 (11.8)
BMI (mean ± SD)	27.0 ± 4.75	
Age at asthma onset, years (mean ± SD)	32.94 ± 12.39	
Disease duration, years (mean ± SD)	14.37 ± 7.48	
Follow‐up duration, months, median (min–max)	33 (6–72)	
Biologic treatment, *n* (%)	Omalizumab	10 (19.6)
	Mepolizumab	34 (66.7)
	Benralizumab	7 (13.7)

*Note:* Data are presented as *n* (%) unless otherwise indicated.

Abbreviations: BMI, body mass index; SD, standard deviation.

^a^ Other: Fibromyalgia, Atopic dermatitis, Euthyroid Multinodular Goiter, Urolithiasis.

The asthma phenotypes were non‐allergic eosinophilic 23 (45.1%), allergic eosinophilic 20 (39.2%), and allergic non‐eosinophilic 8 (15.7%). Blood eosinophil counts exceeded 300 cells/μL in 84.3% (*n*: 43) of patients (mean 1027.3 ± 930.0). Seventeen (33.3%) patients had allergic rhinitis and 28 (54.9%) chronic rhinosinusitis with nasal polyps (CRSwNP). Hypertension was present in 12 (23.5%) and hypothyroidism in 5 (9.8%) patients.

Baseline demographic characteristics were assessed for the whole cohort (*n* = 51). Of the seven benralizumab‐treated patients, five had received biologic therapy for less than 12 months and were therefore not candidates for step‐down; the remaining two, although treated for ≥ 12 months, did not undergo step‐down (one owing to a recent exacerbation and one to an ACT score < 20) and were included in the non–step‐down group. After excluding one further patient owing to insufficient follow‐up, between‐group comparisons were performed in 45 patients. Step‐down intervention (discontinuation of add‐on therapy and/or ICS dose reduction) was attempted in 21 of these patients (46.7%; 95% CI, 32.9%–61.0%) and was successful in 17 (81.0%; 95% CI, 60.0%–92.3%) (Figure 2).

**FIGURE 2:**
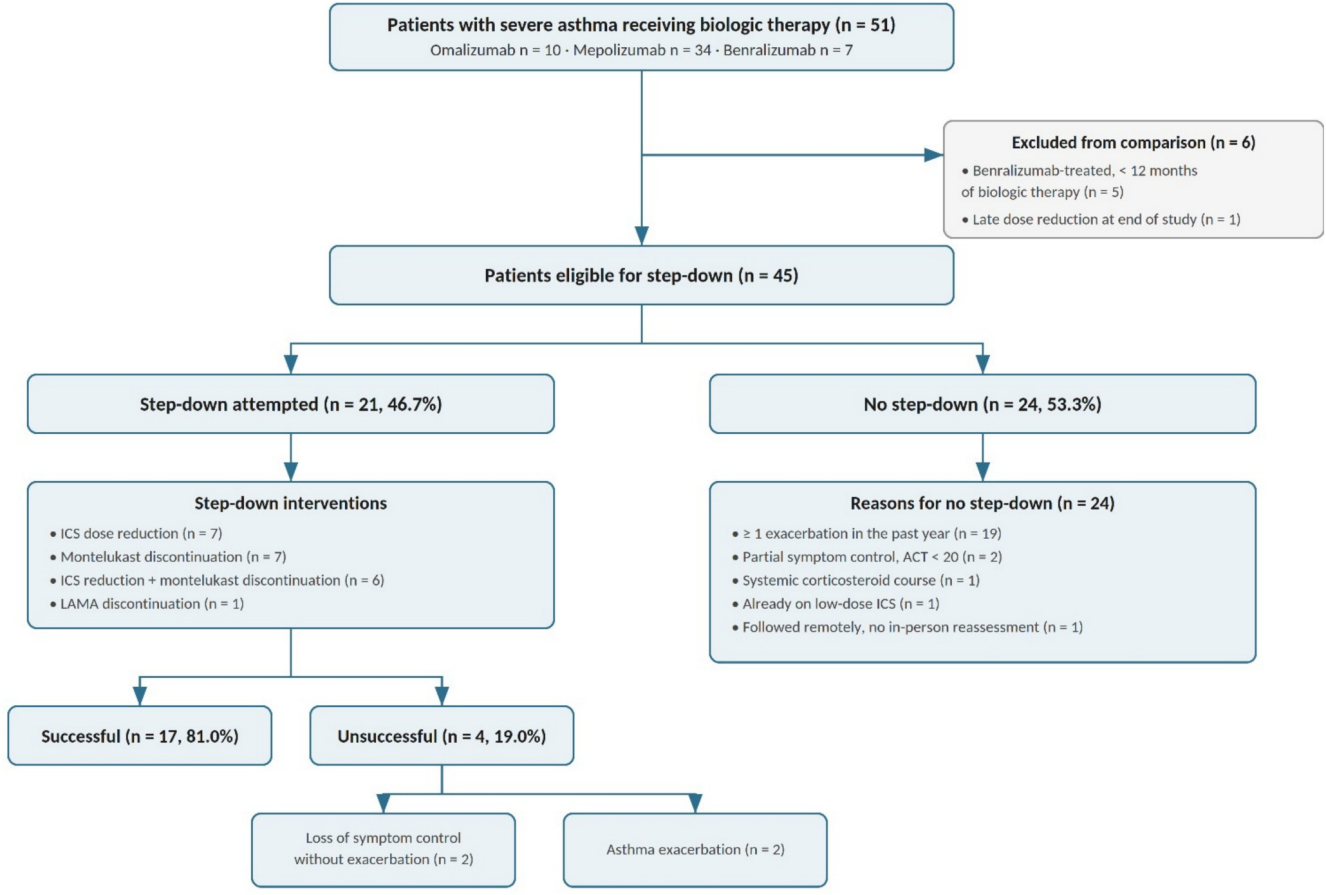
Distribution of patients who applied ICS dose reduction and discontinuation of additional treatments.

### 3.2 Baseline Treatment Characteristics Before Step‐Down

Before step‐down, 8 (38.1%) patients used high‐dose ICS/LABA, 4 (19.0%) high‐dose ICS/LABA + montelukast, 6 (28.6%) medium‐dose ICS/LABA + montelukast, 2 (9.5%) medium‐dose ICS/LABA, and 1 (4.8%) high‐dose ICS/LABA + montelukast + LAMA (Figure 3). Some patients were receiving medium‐dose ICS/LABA at the time of the step‐down assessment; these patients had been started on biologic therapy because their asthma had worsened on previous reduction of controller treatment, thereby fulfilling the definition of severe asthma.

**FIGURE 3:**
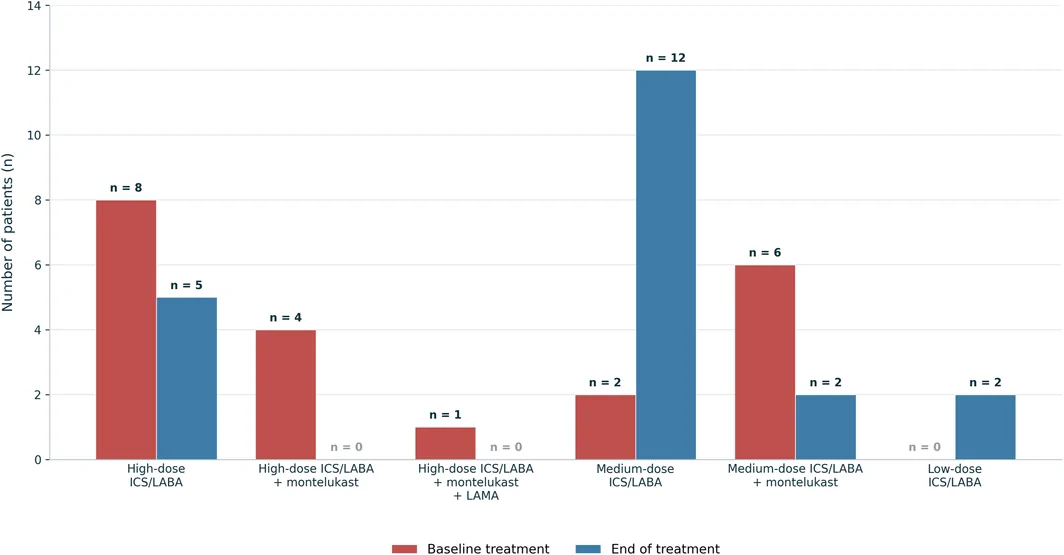
Distribution of drugs used before and after dose reduction attempt.

### 3.3 Step‐Down Interventions

During follow‐up, ICS dose reduction and montelukast discontinuation were implemented as separate interventions in distinct patient subgroups: ICS dose was reduced as the sole intervention in 7 (33.3%) patients, and montelukast was discontinued as the sole intervention in 7 (33.3%) patients. Both interventions (ICS dose reduction and montelukast discontinuation) were carried out simultaneously in a separate group of 6 (28.6%) patients.

LAMA was discontinued in 1 (4.8%) patient. At the final visit, 12 (57.1%) patients were on medium‐dose ICS/LABA, 5 (23.8%) on high‐dose ICS/LABA, 2 (9.5%) on medium‐dose ICS/LABA + montelukast, and 2 (9.5%) on low‐dose ICS/LABA (Figure 3). Mean follow‐up after initiation of step‐down was 8.15 ± 5.23 (2–15) months. Clinical characteristics of the patients who underwent step‐down intervention are presented in Table 2.

**TABLE 2:** Longitudinal outcomes of patients who underwent step‐down intervention (*n* = 21).

Parameter	*n*	At step‐down visit	At last visit	*p* value
ACT score	21	23.90 ± 1.22	23.76 ± 1.76	0.723
FEV1, L	8	3.06 ± 0.86	2.75 ± 0.82	0.230
FEV1, % predicted	7	86.71 ± 21.73	80.43 ± 13.20	0.458
FEV1/FVC, %	7	75.29 ± 8.75	76.57 ± 8.54	0.729

*Note:* Comparisons were performed using the paired‐samples *t*‐test. Patients had received biologic therapy for a mean of 42.6 ± 14.0 months, with step‐down initiated after a mean of 34.2 ± 12.6 months.

Abbreviations: ACT, asthma control test; FEV1, forced expiratory volume in 1 second; FVC, forced vital capacity.

### 3.4 Unsuccessful Step‐Down Cases

Step‐down was unsuccessful in four patients (19.0%), all of whom had an eosinophilic non‐allergic phenotype, were receiving mepolizumab, and were maintained on medium‐dose ICS/LABA (one additionally on montelukast); two of them had comorbid CRSwNP. During post–step‐down follow‐up, two patients lost symptom control (ACT 23 → 19 and 25 → 18) without exacerbation, necessitating a return to the previous dose, whereas the other two developed asthma exacerbations. The two patients who exacerbated had higher baseline blood eosinophil counts (1260 and 3170 cells/μL), whereas the two who lost symptom control had lower counts (440 and 380 cells/μL) (Table 3, Figure 2).

**TABLE 3:** Clinical characteristics of the four patients with unsuccessful step‐down.

Variable	Case 1	Case 2	Case 3	Case 4
Sex/age	Male/59	Male/28	Male/67	Female/24
Phenotype	Eosinophilic non‐allergic	Eosinophilic non‐allergic	Eosinophilic non‐allergic	Eosinophilic non‐allergic
Biologic	Mepolizumab	Mepolizumab	Mepolizumab	Mepolizumab
Baseline step	Medium‐dose ICS/LABA	Medium‐dose ICS/LABA	Medium‐dose ICS/LABA	Medium‐dose ICS/LABA + montelukast
ACT (baseline)	25	24	23	25
ACT (post step‐down, at last visit)	25	22	19	18
Exacerbations (previous year)	1 attack	3 attacks	No exacerbation	No exacerbation
Baseline (pre‐biologic) blood eosinophils (/μL)	1260	3170	440	380
Comorbidities	Diabetes mellitus	CRSwNP	Hypertension, allergic rhinitis	CRSwNP

*Note:* Baseline ACT refers to the value at the step‐down visit; post step‐down ACT refers to the value at the last visit. Baseline blood eosinophil counts were measured before initiation of biologic therapy.

Abbreviations: ACT, asthma control test; CRSwNP, chronic rhinosinusitis with nasal polyps; ICS, inhaled corticosteroid; LABA, long‐acting beta‐2 agonist.

### 3.5 Clinical Outcomes After Step‐Down

Although the mean follow‐up duration after treatment step‐down was 8.15 ± 5.23 months, 8 of the 21 patients who underwent treatment reduction were followed for at least 12 months. In 7 of these patients, the reduced treatment regimen was maintained throughout the follow‐up period without the development of treatment failure.

No significant worsening was observed in ACT, FEV1(L), or FEV1/FVC between the step‐down visit and the final visit (*p* = 0.723, *p* = 0.230, and *p* = 0.729, respectively; paired analyses) (Table 2).

### 3.6 Comparison Between Step‐Down and Non–Step‐Down Groups

Step‐down patients were more likely to be male (66.7%, *p* = 0.005) (Table 4). Women had more allergic rhinitis than men (44.0% vs. 15.0%, *p* = 0.037). No other clinical or laboratory differences were observed. There were no significant differences in final FEV1, ACT scores, or eosinophil counts between the step‐down and non–step‐down groups. However, the step‐down group had fewer exacerbations in the previous year (median 0 vs. 1, *p* < 0.001), with no difference in hospitalizations. Nonetheless, because the absence of exacerbations in the preceding year was itself a step‐down eligibility criterion, this difference reflects the selection design rather than an independent association. Follow‐up duration was longer in the step‐down group (44 [13–67] vs. 28 [12–72] months, *p* = 0.007), with a non‐significant trend toward earlier asthma (28.4 vs. 34.8 years, *p* = 0.090).

**TABLE 4:** Comparison of patients with versus without step‐down intervention (*n* = 45).

Variable		Step‐down (*n* = 21)	Non‐step‐down (*n* = 24)	*p*
Gender, *n* (%)	Female	7 (33.3)	18 (75.0)	**0.005**
	Male	14 (66.7)	6 (25.0)	
Age, years		45.05 ± 14.89	47.25 ± 11.15	0.574
Body mass index		25 (23–34)	26 (19–46)	0.945
Age at asthma onset, years		28.43 ± 13.23	34.79 ± 11.42	0.090
Atopy, *n* (%)	Atopic	10 (47.6)	11 (45.8)	0.905
	Non‐atopic	11 (52.4)	13 (54.2)	
Blood eosinophilia ≥ 300 cells/μL, *n* (%)	Eosinophilic	17 (81.0)	20 (83.3)	1.000
	Non‐eosinophilic	4 (19.0)	4 (16.7)	
Smoking, *n* (%)	Non‐smoker	10 (47.6)	15 (62.5)	0.434
	Ex‐smoker	10 (47.6)	7 (29.2)	
	Current smoker	1 (4.8)	2 (8.3)	
Allergic rhinitis, *n* (%)		6 (28.6)	8 (33.3)	0.731
Nasal polyposis, *n* (%)		13 (61.9)	10 (41.7)	0.175
≥ 1 systemic comorbidity, *n* (%)		8 (38.1)	14 (58.3)	0.175
Follow‐up time, months		44 (13–67)	28 (12–72)	**0.007**
Biologic therapy, *n* (%)	Omalizumab	4 (19.0)	6 (25.0)	0.326
	Mepolizumab	17 (81.0)	16 (66.7)	
	Benralizumab	0 (0.0)	2 (8.3)	

*Note:* Within the non–step‐down group (*n* = 24), 23 patients were ineligible for step‐down as they did not meet the predefined eligibility criteria (≥ 1 exacerbation in the preceding year, systemic corticosteroid course, partial symptom control [ACT < 20], or maintenance therapy already at low‐dose inhaled corticosteroid), whereas 1 otherwise‐eligible patient was followed remotely, precluding in‐person reassessment. Two benralizumab‐treated patients who met the ≥ 12‐month criterion but did not undergo step‐down (one owing to a recent exacerbation and one to an ACT score < 20) are included in the non–step‐down group. Continuous variables were compared using the independent‐samples *t*‐test (mean ± SD) or the Mann–Whitney *U* test (median [min–max]) according to the Shapiro–Wilk normality test; categorical variables were compared using the Pearson chi‐square test, Fisher's exact test, or the Fisher–Freeman–Halton exact test, as appropriate. Bold values indicate *p* < 0.05.

### 3.7 Comparison of Biologic Agents Between Groups

Step‐down was attempted in 4 of 10 omalizumab‐treated patients (40.0%) and 17 of 34 mepolizumab‐treated patients (50.0%). Among the 45 patients included in the between‐group comparison, the distribution of biologic agents did not differ significantly between the step‐down and non–step‐down groups (step‐down group: omalizumab, *n* = 4; mepolizumab, *n* = 17; non–step‐down group: omalizumab, *n* = 6; mepolizumab, *n* = 16; benralizumab, *n* = 2; *p* = 0.326, Fisher–Freeman–Halton exact test).

## 4 Discussion

The main finding of this study was that a step‐down intervention was successfully implemented in 81% of patients with severe asthma receiving biologic therapy. These patients maintained the reduced treatment regimen for a mean duration of 8.15 ± 5.23 months without significant deterioration in symptom control, lung function, asthma exacerbations, or the need for systemic corticosteroids. Furthermore, when outcomes at the final examination were compared with those obtained immediately before treatment reduction, no significant changes were observed in ACT scores, FEV1 (L), or the FEV1/FVC ratio. Nonetheless, FEV1 (L) showed a small numerical decline that did not reach statistical significance; given the limited number of patients with paired spirometry (*n* = 8), this finding should be interpreted with caution and confirmed in larger cohorts.

Collectively, these findings support the feasibility and short‐term safety of a supervised treatment reduction strategy in carefully selected patients with well‐controlled severe asthma receiving biologic therapy.

The stability of both symptom control and lung function following treatment reduction is clinically important, given the well‐established adverse effects associated with prolonged exposure to high‐dose inhaled corticosteroids and systemic corticosteroids [1, 23, 24, 25, 26]. Current severe asthma management therefore emphasizes achieving sustained disease control with the lowest effective treatment intensity [1, 2, 17]. In this context, biologic therapies have emerged as key steroid‐sparing agents, allowing optimization of treatment while minimizing corticosteroid‐related burden [12, 27, 28]. Moreover, because reduced adherence to inhaled therapies has been reported in some patients who achieve good disease control under biologic treatment, a structured and supervised step‐down strategy may represent a safer alternative to unsupervised treatment discontinuation [29, 30]. Our findings are consistent with emerging evidence supporting treatment de‐escalation in patients receiving biologic therapy. In the SHAMAL study, ICS dose reduction was achieved in 92% of patients treated with benralizumab, although complete withdrawal of maintenance ICS was associated with a decline in lung function in some patients [17]. Consistent with these findings, treatment in our cohort was reduced to the low‐dose ICS/LABA step without complete ICS withdrawal, resulting in a success rate of 81%.

Similarly, the ANDHI study demonstrated that benralizumab treatment was associated with the discontinuation of several background controller medications, including LAMA (39.4%, *n* = 63/160), LTRA (33.7%, *n* = 69/205), and LABA therapy (11.9%, *n* = 46/387) [31]. Consistent with these observations, montelukast was discontinued in 33.3% (*n* = 7) of patients in our cohort, while both ICS dose reduction and montelukast discontinuation were achieved in 28.6% (*n* = 6). In addition, LAMA therapy was discontinued in 4.8% (*n* = 1) of patients. Taken together, these findings further support the feasibility of treatment simplification and reduction of background controller medications in carefully selected patients receiving biologic therapy.

Evidence specific to omalizumab also supports treatment de‐escalation during biologic therapy. A 2021 real‐world study reported ICS/LABA dose reduction in 40.3% of omalizumab‐treated patients; comparably, ICS dose reduction was applied in 33.3% (7/21) of patients who underwent step‐down in our cohort [32]. Furthermore, a systematic review of 42 studies reported mean reductions in ICS dose of 21.5% after 12 months and 31.9% after 36 months of omalizumab treatment, suggesting that treatment minimization is often a gradual process that becomes increasingly feasible with prolonged disease control [33].

Consistent with this concept, patients who underwent step‐down in our cohort had significantly longer follow‐up durations than those who did not (*p* = 0.007). This finding likely reflects the need for a sustained period of disease stability before treatment reduction can be safely attempted rather than a biologic‐specific effect. However, because of the retrospective single‐center design of the present study, causality cannot be inferred, and this association requires confirmation in larger multicenter cohorts.

Although the number of unsuccessful step‐down cases was small (*n* = 4), these patients exhibited a remarkably homogeneous clinical profile, characterized by a non‐allergic eosinophilic phenotype, treatment with mepolizumab, and, in half of the cases, concomitant CRSwNP. The clustering of these characteristics, all of which are associated with a greater type‐2 inflammatory burden, raises the possibility that certain patients remain partially dependent on maintenance ICS therapy despite biologic treatment and may therefore be more susceptible to loss of disease control following treatment reduction. Interestingly, patients who experienced exacerbations had higher blood eosinophil counts than those who experienced loss of symptom control without exacerbation, suggesting that different mechanisms of treatment failure may exist across the eosinophilic spectrum. Given the small number of events, these findings should be regarded as hypothesis‐generating and require validation in larger prospective studies.

Male sex was more common among patients who underwent step‐down, whereas female patients more frequently had concomitant allergic rhinitis. Previous studies have reported that women with severe asthma often experience poorer symptom control and higher exacerbation‐related healthcare utilization [34]. These sex‐related differences, together with the higher prevalence of allergic rhinitis among women in our cohort, may have contributed to the lower implementation rate of treatment reduction in female patients. In addition, patients undergoing step‐down tended to have an earlier age of asthma onset. Although the difference did not reach statistical significance, this observation is consistent with previous evidence suggesting that patients with childhood‐onset asthma may be more likely to maintain disease control following ICS dose reduction [35].

This study has several strengths and limitations. Its major strength lies in the evaluation of treatment reduction strategies in a real‐world cohort of patients with severe asthma receiving biologic therapy, an area for which evidence remains limited. The real‐world nature of the data enhances the clinical applicability of the findings. However, the retrospective design and relatively small sample size limited our ability to identify independent predictors of step‐down implementation or success. The multiplicity of statistical comparisons also increases the possibility of type I error, and therefore all findings should be considered exploratory and hypothesis‐generating. Furthermore, the mean follow‐up duration after step‐down intervention (8.15 ± 5.23 months) may be insufficient to fully assess the long‐term stability of the step‐down approach in a chronic disease such as asthma. The relatively short follow‐up periods observed reflect the real‐world clinical context of our study, in which step‐down interventions were initiated at varying time points throughout the observation period, with some patients not yet having reached 12 months of follow‐up at the time of data collection. This reflects the timing of the data collection cutoff rather than early treatment discontinuation or loss to follow‐up. Follow‐up periods extending beyond 12 months would be expected to provide more robust evidence regarding the durability of treatment minimization.

Another important limitation is the lack of FeNO and sputum eosinophil measurements. Although peripheral blood eosinophil counts were systematically monitored throughout follow‐up and may partially reflect type‐2 inflammatory activity, FeNO provides complementary information regarding ongoing airway inflammation that cannot be fully captured by blood eosinophilia alone. The absence of FeNO data may therefore have limited our ability to identify biological markers associated with successful treatment reduction [36, 37]. Future prospective studies incorporating FeNO alongside blood eosinophils and other biomarkers may help refine patient selection and improve decision‐making regarding step‐down strategies in severe asthma.

## 5 Conclusion

Our findings add to the growing body of evidence supporting treatment de‐escalation in severe asthma and suggest that a supervised step‐down strategy, as recommended by GINA, may be feasible and safe in carefully selected patients with severe allergic or eosinophilic asthma receiving biologic therapy. Given the well‐recognized adverse effects of long‐term corticosteroid exposure and the substantial burden associated with chronic asthma management, optimizing treatment to the lowest effective intensity remains an important clinical objective. Larger prospective multicenter studies are needed to identify predictors of successful step‐down and to establish evidence‐based treatment reduction protocols. In addition to randomized controlled trials, real‐world studies and patient registries will be essential to evaluate the long‐term effectiveness, safety, and generalizability of step‐down strategies in routine clinical practice.

## Author Contributions

**Nagihan Orhan Özer:** conceptualization, investigation, writing – original draft, methodology, validation, visualization, writing – review and editing, software, formal analysis, project administration, data curation, supervision, resources. **Betül Özdel Öztürk:** conceptualization, investigation, writing – original draft, writing – review and editing, visualization, validation, methodology, software, formal analysis, project administration, data curation, supervision, resources. **Begüm Görgülü Akın:** investigation, conceptualization, writing – original draft, validation, methodology, visualization, project administration, formal analysis, software, data curation, resources, supervision, writing – review and editing. **Ceren Kaplankıran:** investigation, conceptualization, writing – original draft, methodology, visualization, writing – review and editing, software, formal analysis, project administration, data curation, supervision, resources. **Şadan Soyyiğit:** conceptualization, investigation, writing – original draft, writing – review and editing, visualization, methodology, validation, formal analysis, project administration, software, data curation, supervision, resources.

## Funding

The authors have nothing to report.

## Conflicts of Interest

The authors declare no conflicts of interest.

## Data Availability

The data that support the findings of this study are available upon reasonable request from the corresponding author, subject to ethical approval and data protection regulations. The data are not publicly available due to privacy and ethical restrictions.
